# Convolutional Neural Network for Specimen-Invariant Structural Health Monitoring of FRC Under Flexural Loading

**DOI:** 10.3390/s26092788

**Published:** 2026-04-29

**Authors:** George M. Sapidis, Ioannis Kansizoglou, Maria C. Naoum, Nikos A. Papadopoulos, Konstantinos A. Tsintotas, Maristella E. Voutetaki, Antonios Gasteratos

**Affiliations:** 1 Laboratory of Reinforced Concrete and Seismic Design of Structures, Civil Engineering Department, School of Engineering, Democritus University of Thrace, 67100 Xanthi, Greece; mnaoum@civil.duth.gr (M.C.N.); nikpapad@civil.duth.gr (N.A.P.); 2 Department of Occupational Therapy, School of Physical Education, Sport Science and Occupational Therapy, Democritus University of Thrace, 69100 Komotini, Greece; iokansiz@ot.duth.gr; 3 Department of Information and Electronic Engineering, Faculty of Engineering, International Hellenic University, 57400 Thessaloniki, Greece; tsintotas@ihu.gr; 4 Structural Science and Technology Division, Architectural Engineering Department, School of Engineering, Democritus University of Thrace, 67100 Xanthi, Greece; mvouteta@arch.duth.gr; 5 Laboratory of Robotics and Automation, Department of Production and Management Engineering, School of Engineering, Democritus University of Thrace, 67100 Xanthi, Greece; agaster@pme.duth.gr

**Keywords:** structural health monitoring (SHM), flexural damage identification, repeated loading, deep learning, convolutional neural network (CNN), electromechanical impedance (EMI), fiber-reinforced concrete (FRC), piezoelectric lead zirconate titanate (PZT)

## Abstract

Reinforced Concrete (RC) structures experience progressive degradation over their service life due to mechanical loading and environmental exposure, leading to reduced bearing capacity and compromised structural safety. Incorporating discrete fibers into concrete mitigates crack propagation and enhances ductility, resulting in fiber-reinforced concrete (FRC) with superior fracture energy, durability, and sustainability characteristics. Despite these advantages, research on Structural Health Monitoring (SHM) techniques for FRC elements remains limited. The Electromechanical Impedance (EMI) method, which exploits piezoelectric transducers as both actuators and sensors, offers high sensitivity for detecting early-stage damage by monitoring variations in local mechanical impedance. This study investigates the effectiveness of a deep learning-enabled EMI framework for assessing the structural condition of FRC beams under flexural loading. A one-dimensional convolutional neural network (1D-CNN) is proposed to automatically extract salient features from high-frequency EMI signatures and classify structural health into three predefined states. The model is rigorously evaluated using specimen-invariant validation to ensure generalization across different FRC specimens, addressing a critical limitation of conventional cross-validation approaches in SHM research. Experimental tests on FRC beams instrumented with surface-bonded PZT transducers provide a dataset of 264 EMI responses for training and validation, enabling direct comparison between common and specimen-invariant validation schemes. The results demonstrate the superior robustness of the specimen-invariant approach and confirm the capability of the proposed 1D-CNN to identify flexural damage progression in FRC elements accurately. An ablation study further highlights the contribution of each architectural component to overall model performance. The findings underscore the potential of integrating EMI-based sensing with advanced deep learning models for reliable, automated, and scalable SHM of next-generation resilient concrete infrastructures.

## 1. Introduction

Reinforced concrete (RC) structures gradually deteriorate throughout their operational lifespan due to exposure to diverse load conditions and various environmental factors [1]. As a result, the bearing capacity of RC structures decreases over time, potentially undermining their structural integrity and occupant safety [2]. The low tensile strength of concrete and its inherently brittle characteristics are well-documented in the existing literature. Consequently, microcracks initiate in areas experiencing tensile stress. Eventually, these microcracks propagate and expand into localized macrocracks, ultimately diminishing the load-bearing capacity of the structural member [3]. Including discrete fibers in concrete significantly enhances its crack propagation resistance. This improvement reduces the material’s susceptibility to damage, increasing its tensile strength and ductility [4,5,6].

Fiber-reinforced concrete (FRC) demonstrates enhanced fracture energy capacities and strain-softening behavior due to the fibers’ ability to effectively inhibit the formation and propagation of macrocracks [7,8]. Furthermore, using synthetic fibers in FRC significantly enhances its corrosion resistance, shrinkage control, and fire safety [9,10]. Additionally, the recyclability of these materials is in alignment with fostering the development of resilient and sustainable societies that prioritize using renewable materials while minimizing ecological impact [11,12].

Lately, feasible Structural Health Monitoring (SHM) techniques for civil engineering applications have thrived. Engineers employ SHM techniques for continuous structural integrity assessment of newly erected and existing structures [13]. The prompt identification of crack formation in essential structural components is vital for mitigating potential loss of bearing capacity and preventing sudden structural failures [14]. Smart piezoelectric materials, like lead zirconate titanate (PZT), have been widely used in SHM [15]. The primary advantages of piezoelectric materials include their versatility as both actuators and sensors, attributed to the piezoelectric effect. Additionally, these materials are available in a wide range of shapes and sizes, making them suitable for various applications. Their cost-effectiveness and durability and reliability further enhance their appeal for integration in numerous technologies [16,17]. Piezoelectric materials have been the basis for several SHM techniques such as Guided Waves (GWs) [18,19], Acoustic Emissions (AEs) [20], ultrasonic [21], and Electromechanical Impedance (EMI).

In contrast with other SHM techniques, EMI employs piezoelectric transducers simultaneously as actuators and sensors, making it a promising SHM technique. Owing to the high-frequency excitations, the EMI technique demonstrates exceptional sensitivity and efficiency in rapidly detecting emerging damage. The EMI technique operates on the principle that a structure’s mechanical impedance affects the PZT patches’ EMI response. As a result, crack formation degrades the structural parameters, such as mass, stiffness, and damping, of the material near the PZT transducers, and it can be detected as an alteration in its EMI response. The literature indicates that PZT transducers can be either epoxy-bonded to the external surface of the RC member [22,23] or incorporated as embedded smart aggregates [24]. Recent studies have highlighted the effectiveness of EMI-based SHM for RC structural elements. The existing literature has explored the identification of flexural damage in ordinary RC beams [25,26] and pre-stressed concrete beams [27,28,29]. Additionally, the efficacy of the EMI technique in the rapid diagnosis of brittle failures in shear-deficient RC beams [24,30] and in shear-critical sub-assemblies of RC structures, such as beam–column joints [22,31,32,33], has been well-documented. While considerable attention has been paid to PZT-enabled EMI-based SHM for RC members, the discussion surrounding the monitoring of FRC remains relatively limited [22,34].

The advent of Artificial Intelligence (AI) and, more specifically, advanced machine learning algorithms has revolutionized data acquisition, processing, and interpretation across diverse fields such as industry [35], healthcare [36], robotics [37], and structural engineering applications [22,38]. Recent studies have demonstrated the growing role of machine learning in structural engineering applications, such as the development of data-driven models for seismic fragility assessment of hollow-core bridge piers and the prediction of shear capacity in reinforced concrete piers [39,40]. Deep learning approaches, in particular, have exhibited exceptional capabilities in identifying complex, nonlinear patterns embedded within large-scale datasets that are often challenging to capture using conventional statistical methods [41,42]. When integrated with SHM, AI techniques facilitate automated crack detection [43], damage localization [44], and the prediction of degradation trends, thereby enhancing decision-making processes for preventive maintenance and safety management [45]. More specifically, recent studies have explored the use of deep learning techniques, particularly one-dimensional convolutional neural networks (1D CNNs), for EMI analysis or admittance signatures in concrete SHM. As an instance, a 1D CNN model has been introduced to perform deep learning directly on raw impedance signals obtained from smart aggregates embedded in concrete specimens, enabling stress monitoring under different loading conditions [46]. Similarly, another study [47] employed 1D CNNs to analyze raw EMA signatures for automated detection of small-scale damages in concrete structures, demonstrating improved accuracy compared to traditional neural network approaches and eliminating the need for complex signal preprocessing. Recent studies have also explored deep learning frameworks for predicting unknown damage states in concrete structures using EMI signals, such as hybrid CNN-based architectures that directly process raw conductance responses to accurately estimate impact-induced damage in reinforced concrete elements [48,49]. Moreover, the incorporation of AI into SHM frameworks has achieved the acceleration towards autonomous systems capable of real-time monitoring and adaptive responses to evolving structural conditions, ultimately contributing to resilient infrastructure systems [50].

Despite their promising performance, the practical deployment of neural networks (NNs) in SHM necessitates rigorous validation strategies to ensure generalization, robustness, and reliability. Common validation schemes include *k*-fold cross-validation, hold-out validation, and leave-one-out cross-validation, each addressing different challenges related to data availability and model overfitting [51]. Among these, leave-one-out cross-validation is often regarded as the most robust and trustworthy method, as it maximizes the use of available data by iteratively training on all but one sample and testing on the excluded instance, thereby minimizing bias and providing an almost unbiased estimate of model performance [52]. Furthermore, the evaluation of NN-based models extends beyond accuracy, encompassing performance indicators such as sensitivity, specificity, and F1-score, which are critical in balancing false positives and false negatives in structural damage detection [53]. Ultimately, benchmarking against experimental datasets and numerical simulations further strengthens the confidence in AI-driven SHM methodologies.

To address the aforementioned challenges related to reliable damage identification from EMI measurements and the generalization of data-driven models across different structural specimens, this work proposes a deep learning-based SHM framework for FRC beams under flexural loading. More specifically, the main contributions of this study can be summarized as follows:

- We develop a 1D-CNN architecture capable of automatically extracting discriminative features directly from EMI signals, thereby eliminating the need for manual feature engineering in the SHM of FRC beams.
- We introduce and systematically evaluate a specimen-invariant validation scheme that assesses the ability of the model to generalize across different structural specimens. This approach addresses limitations of conventional random cross-validation strategies commonly used in SHM studies and provides a more realistic evaluation of model performance in practical monitoring scenarios.
- We provide a comprehensive comparative analysis between the proposed 1D-CNN framework and conventional machine learning approaches, *viz.*, Support Vector Machine (SVM) [54] and Deep Neural Network (DNN), demonstrating the effectiveness of convolutional feature extraction for EMI-based damage classification.
- We demonstrate the feasibility of integrating EMI-based sensing with advanced deep learning models for automated, scalable, and reliable monitoring of FRC infrastructures.

The remainder of this manuscript is structured as follows: Section 2 presents the methodological framework adopted in this study. First, the principles and implementation of the EMI technique are described, emphasizing its capability for detecting and characterizing structural damage. Subsequently, the architecture of the proposed neural network model is detailed, including its design and configuration for processing high-frequency EMI data and enabling reliable structural health assessment. Section 3 describes the experimental investigation, including the materials and FRC specimens, the flexural loading protocol and data acquisition procedure for SHM, and the specimen-invariant validation strategy employed to assess model generalization. Section 4 presents the experimental results of the proposed damage classification framework. Model performance is evaluated using accuracy metrics and confusion matrices derived from the adopted 4-fold cross-validation scheme, while mean values and corresponding standard deviations are reported to ensure a statistically robust assessment. Finally, Section 5 concludes the paper by summarizing the main findings, discussing the limitations of the present study, and outlining directions for future research.

## 2. Methodology

This section outlines the methodological framework adopted in the present study. First, the principles and implementation of the EMI technique are presented, highlighting its role in detecting and characterizing structural damage. Subsequently, the proposed NN model architecture is described, detailing its design and configuration to process EMI data and allowing for a reliable structural health assessment.

### 2.1. Electromechanical Impedance Technique

The EMI method is typically combined with piezoelectric materials to leverage their direct and inverse piezoelectric phenomenon. The direct piezoelectric effect is the conversion of mechanical stress into electrical charge, while the conversion of applied electrical field into mechanical deformation constitutes the inverse piezoelectric effect. Due to the inherent electromechanical coupling of piezoelectric materials, such as PZTs, the mechanical characteristics of the host structure, where the PZT transducer is attached or embedded, affect the harmonic oscillation of the excited PZT patch. Therefore, any potential degradation in the mechanical properties of the host structure is reflected in the electrical response of a harmonically excited PZT mounted to the structure. In concrete, damage typically manifests as a network of microcracks that gradually expand and coalesce into visible macrocracks, resulting in local degradation of concrete’s mechanical properties. Within the framework of SHM, any conceivable fluctuation in the EMI responses of an affixed PZT sensor signifies the possible development of structural damage nearby. Therefore, for EMI-based prompt damage detection, it is essential to frequently record the EMI responses within a predefined excitation frequency band and to identify damage-induced alterations in the EMI responses.

Recent studies have demonstrated that stress conditions significantly affect both the PZT transducer and the associated host-structure medium in terms of their EMI responses. According to the findings of Naoum et al., the variation in EMI responses due to stress is appreciably greater in embedded PZT sensors than in externally bonded PZT sensors [4]. In addition, studies have shown the impact of stress development and damage in the EMI spectrum of the PZT transducer [24,55]. However, in actual infrastructure, it is impractical to unload the structure to eliminate the influence of applied stress. Therefore, it is essential to establish a procedure to distinguish the influence of developed stress fields from irreversible damage. To address this task, researchers have proposed various methodologies. Zhang et al. have correlated statistical metrics, such as RMSD, MAPD, and CC, to the secant modulus of concrete cubes under monotonic compression loading [56]. Meanwhile, a 2D CNN approach has been introduced for damage detection under varied loading conditions by Ai et al. [57]. In addition, Sapidis et al. present a deep learning approach for the autonomous structural condition assessment of FRC under monotonic and repeated compressive loading, adopting a leave-one-out cross-validation scheme to ensure a more rigorous validation process [22].

### 2.2. Model Architecture

The proposed NN model was designed to process EMI response signals and automatically extract discriminative features for structural damage assessment. Similarly to recent studies, the proposed architecture follows a 1D-CNN structure, which is particularly effective in capturing local temporal patterns within sequential EMI data. The model is composed of three convolutional blocks, each followed by batch normalization (BN) [58] to ensure stable training and mitigate internal covariate shift. The extracted feature maps are subsequently flattened and passed through fully connected layers for higher-level representation learning and classification into three structural condition states. A detailed summary of the architecture is presented in Table 1.

**Figure 1:**
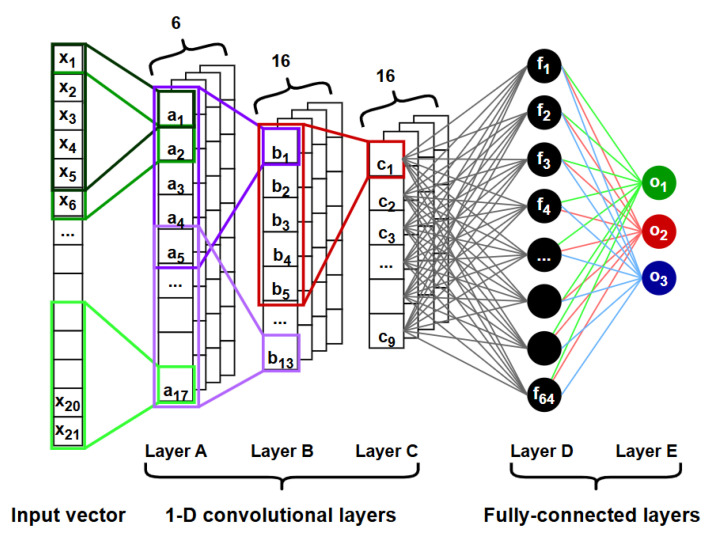
Schematic representation of the proposed 1D-CNN architecture, including convolutional layers with batch normalization, fully connected layers, and the final classification output.

Here, *L* denotes the length of the input sequence. The model concludes with a softmax activation function applied to the final fully connected layer, providing probabilistic predictions for the three predefined structural conditions. A schematic representation of the proposed neural network architecture is illustrated in Figure 1, which complements the tabular description provided above.

The adopted 1D-CNN architecture follows standard convolutional design principles commonly used in signal-processing applications. Its configuration was selected to effectively capture local frequency-dependent patterns within the EMI spectra while maintaining a relatively simple model structure. The final architecture was empirically determined through preliminary experiments in order to balance feature extraction capability, computational efficiency, and training stability given the available dataset’s size.

## 3. Experimental Investigation

This section presents the experimental setup conducted to evaluate the proposed deep learning-based SHM framework for FRC beams under flexural loading. First, the materials and specimen preparation are described, and then the loading protocol and EMI data acquisition procedure are elaborated, as well as the specimen-invariant validation strategy adopted to ensure robust and unbiased assessment of model generalization is discussed.

### 3.1. Materials and Specimens

The experimental study encompasses the casting of four FRC prismatic beams. The FRC was mixed in accordance with the principles of ASTM C192 [59]. In particular, the coarse and fine aggregates were dry-mixed for 30 s in a rotary mixer. The coarse aggregate, with a nominal particle size up to 16 mm, was produced from crushed limestone. Furthermore, a mixture of crushed limestone sand and natural silica was utilized as the fine aggregate. Subsequently, the commercial type II-42.5 N Portland cement was added and mixed for an additional 30 s. Then, 60% of the water was added and mixed for an additional 30 s. Following, the remaining water and a high-range superplasticizer, constituting 0.7% of the cement, were added, to improve workability, and mixed for one additional minute. The mass proportions of cement, water, coarse, and fine aggregate were 1:0.56:2.8:3.3. Finally, the macro-synthetic fibers were incrementally incorporated during stirring to ensure the homogeneity and flowability of the fresh FRC mixture and to prevent fiber agglomeration at a dosage of approximately 5 kg/$m^{3}$. The aspect ratio of the macro-synthetic fibers was 70, with a length of 50 mm and a 0.715 mm equivalent diameter. Furthermore, the macro-synthetic fibers have an elastic modulus of 6 GPa and tensile strength of 430 MPa.

The prismatic beams have a length of 1000 mm, a height of 200 mm, and a width of 150 mm. Furthermore, six standard-cylinder FRC specimens with a 150 mm diameter and 300 mm height were cast to evaluate the mean compressive strength of FRC at 28 days. The specimens were cured at controlled conditions of 20 ± 2 °C and RH > 95% for 28 days. Subsequently, PZT sensors were meticulously adhered to the specimen surface using epoxy resin. The specimens have different configurations of a six-sensor PZT array. However, this study utilizes solely the EMI response of the PZT sensor, which was consistent across all specimens. In particular, a PZT transducer was placed at the midspan of the bottom surface of each specimen, 100 mm from the loading points. The rationale for this decision rests on three pillars. The utilization of a single PZT approach for flexural damage detection constitutes the first pillar. This was facilitated by the development of the critical flexural crack in the midsection of all specimens, despite the incorporation of discrete fibers owing to the four-point loading configuration. The second pillar was to exploit the proximity of the selected PZT transducer to the cracking region. According to the literature, the distance of a PZT sensor to the damage location, characterized by surface cracks in concrete, adversely affects the probability of successful detection. The final pillar pertains to the capability of the proposed SHM scheme to differentiate between damage formation and the impact of induced stresses, which is highly contingent upon the positioning of the PZT sensor. Therefore, only the EMI responses of the PZT fixed sensors on the lower surface in the midspan of each specimen, where the flexural stresses reach their maximum values, were used in this study.

### 3.2. Flexural Loading and Data Acquisition for SHM

**Figure 2:**
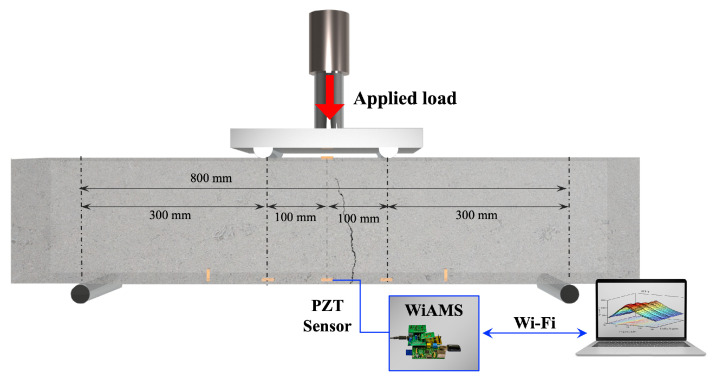
The experimental setup for the bending testing of FRC specimens and the acquisition of PZTs’ EMI responses.

**Figure 3:**
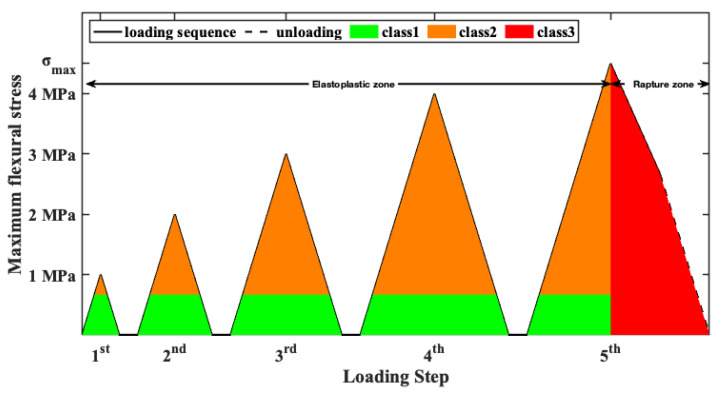
Schematic representation of the flexural loading sequence and the corresponding ranges associated with each EMI response class.

As previously mentioned, the experimental program involves four-point bending of four FRC beams under repeated loading, in accordance with ASTM C1609 [60]. The experimental setup consists of two line loads, each located 100 mm from the mid-plane of the specimens, as illustrated in Figure 2. Furthermore, two hardened-steel roller supports were used to form a span of 800 mm. Thus, two shear spans were formed with a length of 300 mm and a midsection of 200 mm under pure bending. The specimens underwent incremental, repeated bending loading at five various load levels, as shown in Figure 3. In the first four steps of the loading sequence, the flexural stress in the midsection increased by 1 MPa at each step. Then, in the fifth step, the load was increased until the FRC beam reached its maximum bending strength, at which point a fatal bending crack formed in its midsection. The loading rate was kept at approximately 0.9–1.2 MPa/min to ensure sufficient time to acquire a large dataset of EMI responses for each structural condition. Furthermore, at the end of each loading step, the specimen was fully unloaded to measure the EMI responses of the PZT sensors at release conditions.

As mentioned, this research employs a previously established SHM scheme by Sapidis et al., which has been used to autonomously identify compression damage, to monitor FRC under repeated bending loading [22]. Therefore, a custom-made impedance analyzer, known as the “Wireless Impedance/Admittance Monitoring System” (WiAMS), captures the PZTs’ EMI response throughout the loading sequence. The frequency range was set near the PZT patch’s resonant frequency, specifically 90–110 kHz, with a resolution of 1 kHz for the PZT patches used in this study. The application of narrow-frequency-range sweeps facilitates the acquisition of multiple EMI responses throughout the loading sequence of each specimen. This configuration results in a fixed-length feature vector of L=21 points, corresponding to the discrete frequency samples within the selected range. This vector forms the input sequence to the 1D-CNN architecture presented in Section 2.2. Prior to being fed into the model, each input vector is normalized using z-score normalization to ensure numerical stability and improve learning efficiency [61]. The normalization is defined as follows:

(1) $${\hat{x}}_{i}=\frac{x_{i}-\mu }{\sigma },$$

where $x_{i}$ denotes the *i*-th element of the input vector, while μ and σ correspond to the mean and standard deviation of the specific feature, i.e., frequency point, computed across the entire dataset. In other words, for each position *i* in the input vector, normalization is performed using the statistics of that feature across all samples, ensuring consistent scaling among the dataset. This integration enables real-time data collection and analysis, enhancing prompt damage detection.

In contrast to previous studies, this study proposes dividing EMI signatures into three clusters based on the specimen’s structural integrity during the measurement. The division of the class was governed by two principal aspects. The first aspect pertained to the structural condition of the specimen, characterized by the exceedance of the specimen’s bearing capacity and the development of the critical flexural crack, which was categorized into two subgroups: the elastoplastic zone and the rupture zone. The second aspect involved eliminating false alarms attributable to increased stress in the vicinity of the PZT sensor. Thus, the elastoplastic region is classified into two categories based on whether the flexural stress proximal to the PZT patch exhibits 10% of its maximum flexural strength. In particular, the first class consists of measurements captured prior to the development of the fatal bending crack and the negligible bending stresses, lower then 10% of its maximum strength, as shown in Figure 3 in green. Similarly, EMI responses captured prior to the development of the fatal bending crack and under severe bending stresses, higher than 10% of the specimen’s bearing capacity, are labeled as second class and marked in orange in Figure 3. In contrast, the EMI responses recorded during the rupture zone of the specimen, namely after the formation of the critical bending crack, are classified as third class and shown in red in Figure 3. Additionally, a series of EMI signatures was recorded under pristine conditions of the FRC specimens to serve as a baseline. Thus, the proposed SHM scheme aims to identify the formation of a fatal crack, regardless of the specimen’s stress condition, thereby reducing false damage detections and providing useful information on the structural condition of FRC beam specimens. Therefore, the proposed SHM scheme could be utilized in decision-making processes regarding the structural safety of FRC structures, thereby enhancing perception and robustness.

### 3.3. Specimen-Invariant Validation

Considering the above, the dataset consists of 264 EMI measurements collected from four beam specimens, with approximately 75–100 samples per damage class. The samples are relatively balanced across the considered damage states to avoid bias during training. To rigorously evaluate the generalization capability of the proposed model, a specimen-invariant validation strategy was employed. Since the experimental campaign involved four distinct specimens, the dataset was partitioned at the specimen level to eliminate any overlap between training and validation data originating from the same structural element. This approach prevents data leakage and ensures that the model is assessed on entirely unseen specimens, thereby reflecting realistic SHM deployment scenarios.

**Figure 4:**
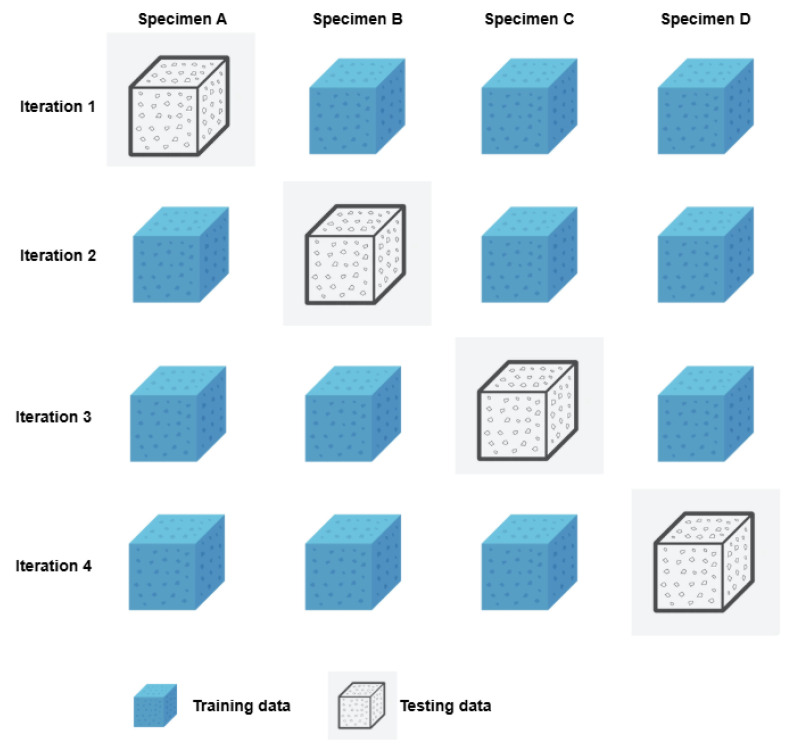
Schematic representation of the specimen-invariant 4-fold cross-validation strategy. In each iteration, one specimen is exclusively used for validation, while the remaining three specimens are used for training, ensuring complete separation between training and validation data at the specimen level.

To that end, a 4-fold cross-validation scheme was implemented, where each fold corresponded to one specimen. In each iteration, data from three specimens were used for training, while the remaining specimen was exclusively reserved for validation. This leave-one-specimen-out rotation was repeated four times, allowing each specimen to serve once as the validation set. The reported performance metrics represent the average results and the standard deviation across the four folds, providing a robust and unbiased estimate of the model’s classification capability for the three predefined structural states. A schematic illustration of the specimen-invariant 4-fold cross-validation strategy is presented in Figure 4, highlighting the rotation of the validation specimen across the four iterations.

### 3.4. Hyperparameter Configuration and Training Setup

The proposed 1D-CNN model was trained using the Adam optimization algorithm [62] with an initial learning rate of 0.001. The categorical cross-entropy loss function was used as the training criterion, which is commonly applied in multi-class classification tasks [63]. To mitigate potential overfitting due to the limited size of the dataset, the network was trained for 100 epochs with a batch size of 32. The hyperparameters were selected based on preliminary experiments to achieve stable convergence and reliable performance of the model. The Adam optimizer was chosen due to its adaptive learning capability and effectiveness in training DNNs [62]. The selected learning rate provided a good balance between convergence speed and training stability.

During training, the model parameters were updated using mini-batch gradient descent. Xavier (Glorot) initialization was employed for the network weights to facilitate stable gradient propagation during training [64]. The training process was monitored using validation data across epochs to ensure proper convergence and to detect potential overfitting. All experiments were implemented using the PyTorch 2.2 deep learning framework [65] and executed on a GPU-enabled computing environment equipped with an NVIDIA Tesla-class GPU. The hyperparameter setup adopted for this study is summarized in Table 2.

## 4. Results

This section presents the experimental results obtained from the proposed damage classification framework. The performance of the developed models is evaluated using accuracy metrics and confusion matrices derived from the adopted 4-fold cross-validation scheme. Mean values and corresponding standard deviations are reported to ensure a comprehensive and statistically robust performance assessment.

### 4.1. Mechanical Behavior of Specimens and EMI Signature Acquisition

**Figure 5:**
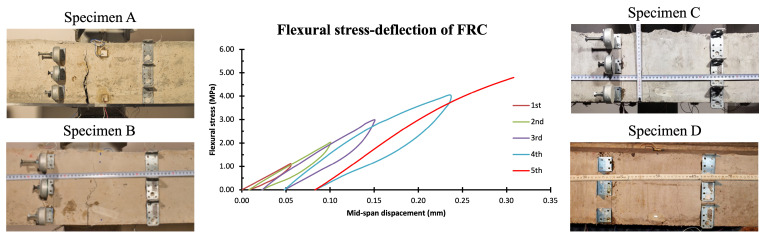
Mechanical behavior and cracking pattern of the FRC specimens at the end of the loading sequence.

The mechanical behavior and the resulting variation in EMI signatures will be discussed in this subsection. As aforementioned, this study involves the experimental investigation of EMI-based damage detection in FRC specimens under flexural loading. Therefore, four FRC beams were subjected to four-point loading and exhibited a typical flexural response and brittle failure, despite the addition of discrete fibers. Figure 5 illustrates the typical mechanical response of the FRC beams concerning flexural stress in relation to midspan deflection. Furthermore, the cracking pattern of the specimens is also illustrated in Figure 5. At the final loading stage, a surface crack developed in the midspan, approximately 50 mm from the central section of the specimens, and it was oriented perpendicular to the longitudinal axis of the beam.

**Figure 6:**
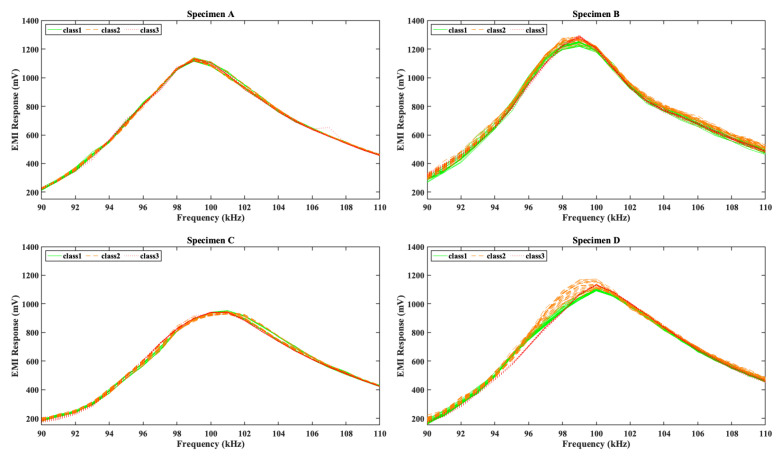
Evolution of the EMI responses of the midspan PZT transducers of the FRC specimens during the loading sequence.

As described in Section 3.2, during the loading sequence, the EMI responses of the midspan PZT transducers were acquired using the WiAMS device. Figure 6 presents the EMI response of the PZT transducer for each specimen within a narrow frequency range of 90–110 kHz. The EMI responses were classified into three distinct categories based on the structural condition of the specimen and the stress fields developed during measurement. The first category comprises EMI responses obtained prior to the initiation of the critical flexural crack, under stress levels below 10% of the specimen’s maximum flexural strength, and is depicted with green lines. The second category includes EMI responses recorded before the formation of the critical flexural crack, but at stress levels exceeding 10% of the maximum flexural strength; these responses are illustrated in orange. The third category corresponds to EMI responses acquired following the formation of the critical crack and the consequent loss of the specimen’s load-bearing capacity, and it is represented with red lines. Based on the observations presented in Figure 6, it can be inferred that the magnitude of variation in the EMI responses, induced by stress and damage evolution, differs among the specimens’ PZT transducers. Nevertheless, all specimens consistently exhibit a stress-induced leftward shift. Furthermore, the formation of the critical crack is associated with a reduction in the bandwidth of the EMI response, in agreement with the findings of recent studies [24,66]. However, the identification of the specimen’s structural condition directly from raw EMI responses remains challenging and prone to error. Therefore, a more robust methodology is required to reliably extract the structural condition of the specimen from the EMI data.

### 4.2. Ablation Study

An ablation study was conducted to assess the effectiveness of the proposed 1D-CNN architecture in comparison with alternative conventional machine learning models. In particular, the classification performance of the proposed model was evaluated against an SVM and a simple DNN with fully connected layers.

**Figure 7:**
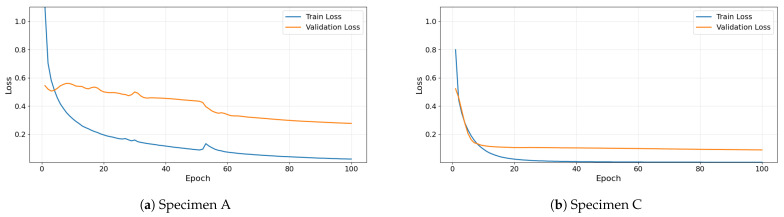
Training and validation loss curves of the proposed 1D-CNN model during training, demonstrating the convergence behavior for Specimens A and C.

For each model, the results are presented for all four folds of the 4-fold cross-validation procedure, including classification accuracy and the corresponding confusion matrices. Additionally, the mean accuracy and standard deviation across the four folds are reported to quantify performance stability and robustness. This comparative analysis enables a systematic evaluation of the contribution of the convolutional feature extraction mechanism and the architectural design choices incorporated into the proposed 1D-CNN model. Representative training and validation loss curves of the proposed 1D-CNN model are presented in Figure 7. The results illustrate the evolution of the loss during the training process for Specimens A and C, demonstrating the convergence behavior of the network across epochs, with no evident overfitting observed.

Table 3 presents the classification performance obtained for each validation specimen under the adopted 4-fold cross-validation scheme, including accuracy (Acc), precision (Prec), recall (Rec), and F1-score (F1) [67], along with the corresponding mean values and standard deviations. The proposed 1D-CNN consistently outperforms both the SVM and the simple DNN across all four specimens, achieving a mean accuracy of 84.15%, compared to 56.84% for the DNN and 54.11% for the SVM. Similar trends are observed for the additional evaluation metrics, where the 1D-CNN attains mean precision, recall, and F1-score values of 86.29%, 84.15%, and 83.43%, respectively, clearly exceeding the corresponding results of the baseline methods.

Notably, the 1D-CNN demonstrates superior robustness, as reflected by its lower standard deviation in accuracy (14.82%), indicating more stable performance across different specimens. In contrast, the SVM and DNN exhibit considerably larger variability (25.12% and 20.75%, respectively), suggesting reduced generalization capability. This trend is also consistent across the additional performance metrics, where the convolutional architecture exhibits lower variability compared to the alternative models. It should be noted that the observed variability across folds is partly attributed to the specimen-invariant validation strategy, where the model is evaluated on an unseen specimen, and to inherent differences in the structural and EMI response characteristics among the tested beams.

Meanwhile, a particularly large performance gap is observed for Specimen C, where the proposed 1D-CNN achieves substantially higher accuracy compared to the SVM and DNN architectures. This behavior can be attributed to the ability of convolutional layers to capture localized patterns and correlations within the EMI spectra across the frequency domain, which are closely related to structural damage evolution. In contrast, the SVM and fully connected DNN rely mainly on global feature representations and may therefore fail to adequately capture such localized spectral variations. Additionally, differences in the EMI response characteristics among specimens may influence the classification difficulty under the specimen-invariant validation scheme. Overall, the results confirm that the convolutional feature extraction mechanism enables the proposed architecture to more effectively capture discriminative patterns in the EMI signals, leading to improved and more reliable damage classification.

**Figure 8:**
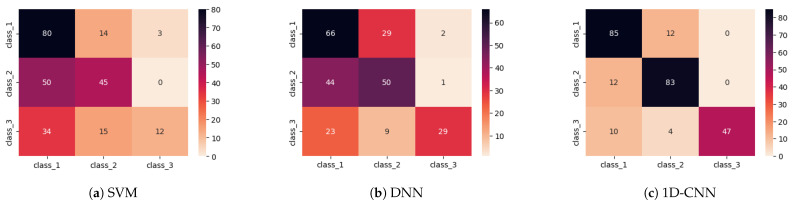
Mean confusion matrices obtained from the 4-fold cross-validation procedure for (**a**) SVM, (**b**) DNN, and (**c**) the proposed 1D-CNN model.

Figure 8 presents the mean confusion matrices obtained from the 4-fold cross-validation for the three evaluated models, *viz.*, SVM, DNN, and the proposed 1D-CNN. The obtained results indicate that the SVM struggles to correctly classify instances of classes 2 and 3, frequently mislabeling them, while the DNN shows noticeable misclassifications in classes 1 and 3. In contrast, the 1D-CNN demonstrates the highest overall classification performance, with strong discrimination across all three classes and minimal misclassification. These results highlight the advantage of the convolutional feature extraction mechanism in capturing complex patterns within the EMI signals, resulting in more robust and reliable structural state identification compared to traditional machine learning and fully connected neural network approaches.

### 4.3. 1D-CNN vs. 2D-CNN

To further evaluate the effectiveness of the proposed 1D-CNN architecture, a comparative analysis with a 2D-CNN architecture has been conducted. Since the original EMI data consist of one-dimensional feature vectors, the 2D-CNN model was implemented by reshaping the input into a pseudo two-dimensional representation, i.e., 3×7, allowing for the application of two-dimensional convolutional operations under the same experimental conditions. Table 4 presents the classification performance of both models in terms of accuracy, precision, recall, and F1-score for each validation specimen, along with the corresponding mean accuracy and standard deviation.

**Figure 9:**
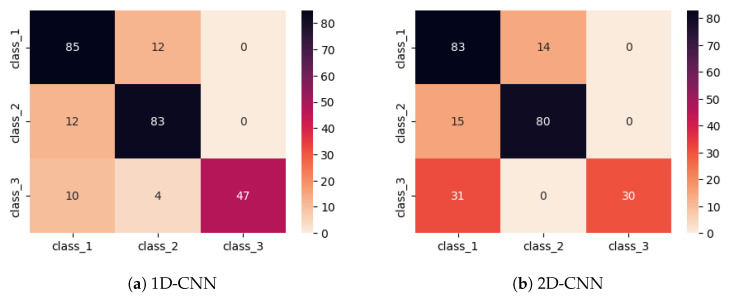
Confusion matrices for the 1D-CNN and 2D-CNN models.

The results indicate that the proposed 1D-CNN consistently outperforms the 2D-CNN across most validation specimens, achieving higher accuracy and improved overall classification metrics. This behavior can be attributed to the fact that EMI signals are inherently one-dimensional, and thus convolution along the original signal dimension allows for the more effective extraction of physically meaningful patterns. In contrast, the 2D representation introduces artificial spatial relationships that do not necessarily correspond to the underlying physics of the problem. To further illustrate the classification behavior, Figure 9 presents indicative confusion matrices obtained for both architectures. Overall, the comparison confirms that the 1D-CNN architecture is better aligned with the nature of EMI data, providing improved performance while maintaining a simpler and more computationally efficient model structure.

### 4.4. Specimen-Invariant vs. Conventional Cross-Validation

This subsection presents a comparative analysis between the specimen-invariant validation strategy and a conventional random split cross-validation approach. Both strategies were implemented using a 4-fold cross-validation scheme, and performance metrics are reported for each fold separately, along with their mean values and standard deviations. Confusion matrices are also provided to offer detailed insight into class-wise prediction behavior and potential misclassification patterns.

The comparison aims to highlight the impact of data partitioning methodology on the estimated classification performance. While the random split strategy allows samples from the same specimen to appear in both training and validation sets, the specimen-invariant approach enforces strict separation at the specimen level. The results therefore illustrate the differences in generalization capability and provide evidence regarding the reliability and realism of each validation protocol.

In Table 5, the comparative results between the specimen-invariant and conventional random split cross-validation strategies are illustrated. As expected, the random split approach yields higher overall performance, achieving a mean accuracy of 93.11% compared to 84.15% for the specimen-invariant strategy. This outcome is logically attributed to the fact that, under random splitting, samples originating from the same specimen appear in both training and validation sets. Consequently, the model is exposed during training to structural patterns that are highly similar to those encountered during validation, leading to optimistic performance estimates.

In contrast, the specimen-invariant validation constitutes a significantly more challenging evaluation task, as the model is required to generalize to entirely unseen specimens. This setup better reflects real-world SHM scenarios, where the trained model must operate on new structural elements with potentially different material properties, boundary conditions, and damage characteristics. Although the mean accuracy is lower under this protocol, it provides a more realistic and trustworthy estimate of generalization capability. Furthermore, the substantially smaller standard deviation observed in the random split strategy (5.16% versus 14.82%) indicates reduced variability across folds, primarily because specimen-specific differences are not included among the performance challenges faced by the algorithm. Therefore, the specimen-invariant approach, despite its increased difficulty, offers a more rigorous and practically relevant assessment of model robustness and is more representative of the conditions expected in real-world SHM deployment.

**Figure 10:**
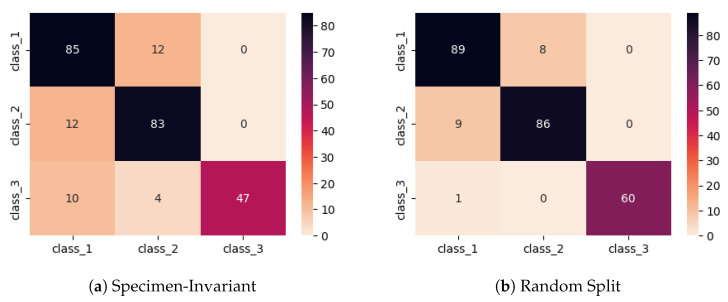
Mean confusion matrices of the proposed 1D-CNN model under (**a**) specimen-invariant cross-validation and (**b**) conventional random split cross-validation.

Figure 10 illustrates the mean confusion matrices obtained under the specimen-invariant and random split validation strategies. The confusion matrix corresponding to the random split approach demonstrates a highly balanced classification performance across all three structural classes, with consistently strong diagonal dominance and limited misclassifications. This uniform behavior across classes and specimens reflects the model’s exposure during training to data originating from the same structural elements that appear in the validation set, thereby reducing the variability and complexity of the classification task.

In contrast, the specimen-invariant strategy presents a more heterogeneous distribution of predictions, with certain classes exhibiting increased confusion. This behavior highlights the presence of intrinsic divergences among different specimens, such as variations in material properties, boundary conditions, and damage evolution patterns, which are not captured under random splitting. While the random split approach yields more visually balanced and higher overall performance, it inherently underestimates the challenges associated with real-world deployment. Therefore, the specimen-invariant confusion matrix provides a more realistic representation of the model’s ability to generalize across structurally distinct conditions.

## 5. Conclusions

This study presented a deep learning-based SHM framework for the classification of damage states in FRC beams using the EMI technique. For this purpose, a 1D-CNN architecture was developed to process EMI signals and automatically extract discriminative features associated with three structural conditions. The introduced model was systematically evaluated through a 4-fold cross-validation scheme and compared against an SVM and a DNN architecture. The experimental dataset comprises 264 individual EMI measurements collected from four FRC beam specimens, where a significant experimental effort was devoted to acquiring a large number of measurements per damage state, i.e., approximately 75–100 samples per class. This provided sufficient variability in the recorded EMI responses for training and validation of the proposed model. The results demonstrated the clear superiority of the 1D-CNN, which achieved the highest mean accuracy and the lowest performance variability across specimens. Furthermore, the comparative analysis between specimen-invariant and conventional random split validation strategies highlighted the importance of rigorous evaluation protocols, revealing that specimen-invariant validation provides a more realistic estimate of generalization capability.

Despite the promising results, certain limitations should be acknowledged. First, contemporary DL applications in various research fields utilize large-scale benchmarks to evaluate their performance and efficiency. However, the field of SHM itself poses, in general, significant barriers regarding collection of experimental data from multiple specimens, and as a result, such a well-established benchmark is yet to be introduced. The abovementioned limitation remains as an open research challenge in the field of SHM since the purpose of the current work does not address this requirement. On the other hand, the exploited dataset is of comparable size to those reported in the literature, placing it in line with the state of the art.

Secondly, to further evaluate the generalization capability of the approach, a specimen-invariant validation strategy was adopted, ensuring that the model was tested on data originating from an unseen physical specimen. Nevertheless, the relatively small number of specimens limits the diversity of structural and environmental conditions represented in the dataset. Future work will therefore focus on expanding the experimental campaign with additional specimens and damage scenarios to further strengthen the statistical robustness and deliver an even higher-quality dataset.

Additionally, the investigation focused on controlled laboratory conditions, and potential sources of variability such as environmental noise, temperature fluctuations, and long-term degradation effects were not explicitly incorporated. These factors may influence EMI measurements in real-world SHM applications and should be carefully considered in future implementations. More specifically, in practical SHM deployments, EMI measurements may be influenced by environmental and operational factors such as temperature variations, humidity, and measurement noise. These effects may alter the impedance signatures and potentially impact the performance of data-driven models if not properly considered. Although the controlled laboratory conditions adopted in this study allow for a clear evaluation of the proposed methodology, future work should investigate the robustness of the approach under varying environmental conditions.

Considering the above, potential research directions could include expanding the experimental campaign to a broader range of structural configurations and damage scenarios to further validate model robustness. The integration of advanced deep learning strategies, such as attention mechanisms or hybrid CNN–recurrent architectures, may improve temporal feature extraction from EMI signals while increasing the necessity for a larger amount of training data. Moreover, incorporating domain adaptation or transfer learning techniques could enhance the model’s ability to generalize across different structural typologies and operational conditions. Finally, real-time implementation within embedded SHM systems and validation under field conditions would constitute a critical step toward practical deployment in resilient and intelligent infrastructure monitoring frameworks.

## Figures and Tables

**Table 1:** Summary of the proposed 1D-CNN model architecture.

Layer	Type	Input → Output	Kernel/Stride	Output Size
1	Conv1d + BN	1 → 6	5 / 1	[6, *L*–4]
2	Conv1d + BN	6 → 16	5 / 1	[16, *L*–8]
3	Conv1d + BN	16 → 16	5 / 1	[16, *L*–12]
4	FC + BN	144 → 64	–	[64]
5	FC	64 → 3	–	[3]

*L* denotes the length of the input sequence. BN: Batch normalization.

**Table 2:** Training hyperparameters of the proposed 1D-CNN model.

Parameter	Value
Epochs	100
Batch size	32
Optimizer	Adam
Learning rate	0.001
Loss function	Cross-Entropy
Weight initialization	Xavier (Glorot)

**Table 3:** Classification performance (%) per specimen using 4-fold specimen-invariant cross-validation.

	SVM				DNN				1D-CNN			
Validation Specimen	Acc	Prec	Rec	F1	Acc	Prec	Rec	F1	Acc	Prec	Rec	F1
Specimen A	44.83	20.10	44.83	27.75	44.83	78.14	44.83	35.66	63.79	69.64	63.79	60.91
Specimen B	46.43	34.22	46.43	36.07	78.57	79.11	78.57	78.71	83.93	85.28	83.93	83.97
Specimen C	34.25	11.73	34.25	17.47	34.25	11.73	34.25	17.47	90.41	91.69	90.41	90.35
Specimen D	90.91	92.32	90.91	90.79	69.70	80.91	69.70	66.69	98.49	98.55	98.48	98.49
**Mean**	54.11	39.59	54.11	43.02	56.84	62.47	56.84	49.63	84.15	86.29	84.15	83.43
**Standard Deviation **	25.12	31.79	25.12	31.04	20.75	32.05	20.75	27.02	14.82	11.64	14.82	15.62

**Table 4:** Classification performance (%) per specimen using 4-fold specimen-invariant cross-validation for 1D-CNN and 2D-CNN.

	1D-CNN				2D-CNN			
Validation Specimen	Acc	Prec	Rec	F1	Acc	Prec	Rec	F1
Specimen A	63.79	69.64	63.79	60.91	62.07	55.49	62.07	55.57
Specimen B	83.93	85.28	83.93	83.97	76.79	77.72	76.79	74.14
Specimen C	90.41	91.69	90.41	90.35	67.12	83.23	67.12	59.78
Specimen D	98.49	98.55	98.48	98.49	98.49	92.32	90.91	90.79
**Mean**	84.15	86.29	84.15	83.43	76.12	77.19	74.22	70.57
**Std. Dev. **	14.82	12.57	14.82	14.53	16.12	13.95	11.08	13.70

**Table 5:** Classification accuracy (%) comparison between specimen-invariant and conventional random split cross-validation strategies.

Validation Specimen	Specimen-Invariant (%)	Random Split (%)
Specimen A	63.79	92.16
Specimen B	83.93	98.00
Specimen C	90.41	86.28
Specimen D	98.49	96.00
**Mean **	84.15	93.11
**Standard Deviation **	14.82	5.16

## Data Availability

The data generated and analyzed during the current study are not publicly available as they are part of an ongoing research project and due to technical and time limitations related to data curation and storage, but they are available from the corresponding author upon reasonable request.

## Abbreviations

The following abbreviations are used in this manuscript:

RC	Reinforced Concrete
FRC	Fiber-Reinforced Concrete
SHM	Structural Health Monitoring
PZT	Piezoelectric lead Zirconate Titanate
EMI	Electromechanical Impedance
CNN	Convolutional Neural Network
SVM	Support Vector Machine
DNN	Deep Neural Network
